# Development and validation of a new microplate assay that utilises optical density to quantify the antibacterial activity of honeys including Jarrah, Marri and Manuka

**DOI:** 10.1371/journal.pone.0243246

**Published:** 2020-12-09

**Authors:** Kathryn J. Green, Kenneth Dods, Katherine A. Hammer

**Affiliations:** 1 School of Biomedical Sciences, The University of Western Australia, Crawley, Western Australia, Australia; 2 CRC for Honey Bee Products, The University of Western Australia, Crawley, WA, Australia; 3 ChemCentre, Resources and Chemistry Precinct, Bentley, Western Australia, Australia; University of Hong Kong, HONG KONG

## Abstract

The phenol equivalence assay is the current industry-adopted test used to quantify the antibacterial activity of honeys in Australia and New Zealand. Activity is measured based on the diffusion of honey through agar and resulting zone of growth inhibition. Due to differences in the aqueous solubilities of antibacterial compounds found in honeys, this method may not be optimal for quantifying activity. Therefore, a new method was developed based on the existing broth microdilution assay that is widely used for determining minimum inhibitory concentrations (MICs). It utilises the four organisms *Staphylococcus aureus* ATCC 29213, *Enterococcus faecalis* ATCC 29212, *Escherichia coli* ATCC 25922 and *Pseudomonas aeruginosa* ATCC 27853, and an optical density endpoint to quantify bacterial growth. Decreases in bacterial growth in the presence of honey, relative to the positive growth control, are then used to derive a single value to represent the overall antibacterial activity of each honey. Antibacterial activity was quantified for a total of 77 honeys using the new method, the phenol equivalence assay and the standard broth microdilution assay. This included 69 honeys with undisclosed floral sources and the comparators Manuka, Jarrah (*Eucalyptus marginata*), Marri (*Corymbia calophylla*), artificial and multifloral honey. For the 69 honey samples, phenol equivalence values ranged from 0–48.5 with a mean of 34 (% w/v phenol). Mean MICs, determined as the average of the MICs obtained for each of the four organisms for each honey ranged from 7–24% (w/v honey). Using the new assay, values for the 69 honeys ranged from 368 to 669 activity units, with a mean of 596. These new antibacterial activity values correlated closely with mean MICs (R^2^ = 0.949) whereas the relationship with phenol equivalence values was weaker (R^2^ = 0.649). Limit of detection, limit of quantitation, measuring interval, limit of reporting, sensitivity, selectivity, repeatability, reproducibility, and ruggedness were also investigated and showed that the new assay was both robust and reproducible.

## Introduction

Honey produced by the European honeybee *Apis mellifera* is well known to have antimicrobial activity. This activity is attributed to a range of factors including high osmolarity, the relatively low pH, the production of hydrogen peroxide by the enzyme glucose oxidase and the action of plant-derived compounds such as flavonoids and phenolic acids [1–3]. Whilst high osmolarity and low pH are factors that are common to almost all honeys, other factors differ according to each floral source. Levels of hydrogen peroxide, and the quantities and types of phenolic compounds present in honeys derived from different floral sources can vary widely, and this directly impacts the characteristics of each honey, including taste and colour, and the level of antibacterial activity [2, 4].

Honey has a range of properties that make it a potentially useful therapeutic agent, health supplement or functional food [5]. Whilst several of these potential benefits require additional rigorous research to validate their usefulness, it is important to be able to accurately quantify a range of characteristics within honeys *in vitro*, including prebiotic activity [6, 7], anti-oxidant activity [8, 9] and antimicrobial activity [10]. In Australia and New Zealand the current industry-adopted commercial test for quantifying the antibacterial activity of honey is known as the phenol equivalence assay, and has also been referred to as the Unique Manuka Factor (UMF) assay [1, 11]. This assay is used by both commercial testing facilities and researchers [10, 12, 13] to quantify antibacterial activity. It was first described in the early 1990s [14] and was adapted from a method published by the New Zealand Dairy industry in 1982 used for detecting inhibitory substances in dairy products. The assay quantifies antibacterial activity using an agar diffusion type of assay. In brief, solutions of 25% honey and a series of phenol solutions are added to wells cut into nutrient agar that has been seeded with the test organism *Staphylococcus aureus* ATCC 25923 [14]. After incubation, the resulting zones of bacterial growth inhibition are compared to zones obtained for the series of phenol standard solutions that have been plotted to generate a standard curve, resulting in a phenol equivalence (PE) value for each honey. The assay can be used to quantify “total activity” (TA) by testing honey alone, or to quantify “non-peroxide activity” (NPA) after the addition of catalase to each honey solution to remove hydrogen peroxide activity. This residual, or non-peroxide activity, is most commonly found in honeys derived from *Leptospermum* species, and as such has also been referred to as the Unique Manuka Factor or UMF [10, 15, 16]. This trademarked measurement has been used extensively in the marketing of Manuka honeys.

Whilst antimicrobial activity methods that are based on agar diffusion, including the PE assay, have clear benefits in terms of being relatively quick, easy and inexpensive, they are not without significant drawbacks. For example, the validity and appropriateness of agar diffusion methods for quantifying antimicrobial activity has been questioned [17, 18]. A commonly-raised criticism is that because agar is a water-based matrix, non-polar antimicrobial compounds (such as those commonly found in many natural products) or antimicrobial compounds with higher molecular weights may not diffuse readily through the agar [1, 18–20]. For example, the phenolic compounds sinapic acid and hesperitin, which have been identified in Jarrah honey (from *Eucalyptus marginata*) [21], are only sparingly soluble in aqueous solvents [22, 23]. This means that the zone sizes obtained for different compounds are not always directly comparable, and that a smaller zone size does not necessarily equate to a less active compound. A further consideration is that agar diffusion methods are widely regarded as generating qualitative data only [18, 20, 24], and that quantitative data can only be produced using dilution type methods, such as broth dilution assays.

In addition to these broader methodological issues, there are a number of limitations and issues that are specific to the PE assay. Firstly, the assay has a relatively high detection threshold and is not particularly sensitive, as illustrated in several previous studies that reported undetectable activity for considerable numbers of honeys. Examples include a large study examining 477 honeys where activity was undetectable in 40% of samples [10], a study of 345 honeys where activity was not detected in 36% of samples [14] and a smaller study investigating 17 honeys where activity was undetectable in 65% of samples [12]. Ideally, an antimicrobial assay should have the capacity to detect activity in all honey samples, given that all honeys when tested at high enough concentrations exert antibacterial activity due to high osmolarity [25]. Secondly, the PE assay assesses activity against only one test organism (*S*. *aureus*), and as such may not provide a full picture of the antibacterial activity profile of a honey. These issues, in addition to others mentioned in the discussion, provided the impetus for the development of a new assay for quantifying the antibacterial activity of honey.

Consultation of published literature shows that a range of methods other than agar diffusion have been used for quantifying the antimicrobial activity of honey. These include those developed for the testing of disinfectant and antiseptics, such as suspension tests [26] and carrier tests [27], and those utilised for the in vitro testing of antibiotics such as agar dilution [28], broth dilution to determine MICs [16] and spectrophotometric methods to characterise bacterial growth inhibition [29, 30]. In particular, broth microdilution methods have been used extensively for testing a broad range of antimicrobial agents, including both conventional antibiotics [31] and novel agents [18]. The output of this assay is the MIC, which is typically defined as the lowest concentration of the agent that completely inhibits or prevents the growth of the test organism [31]. The broth microdilution method has also been used previously for quantifying the antimicrobial activity of honey [32–34]. It is relatively easy to perform and provides quantitative results. However, a limitation of the assay is that a typical doubling dilutions series (e.g. 32, 16, 8, 4, 2%) is unlikely to be sufficient for discriminating between differing levels of activity amongst honeys. In addition, the assay can be both laborious and time consuming to set up, which may not be appropriate for use in a commercial testing laboratory. Lastly, difficulties may be encountered when visually interpreting the MIC, as indistinct or “trailing” endpoints for honeys can occur due to partial growth inhibition [21, 29, 35].

Given the issues and limitations associated with both the existing PE method, and the broth method for determining MICs, the purpose of this research was to develop, assess and validate a new assay for quantifying the antibacterial activity of honey. The assay was developed so that the output or endpoint is a single numerical value to represent to activity of each honey, based on activity shown against four different bacterial test organisms. The new assay is a modification of the broth microdilution assay, and utilises a spectrophotometric optical density endpoint rather than relying on a subjective, visually determined endpoint. The optical density values are then used to calculate a single antibacterial activity value. This new assay offers a potential replacement for the currently used phenol equivalence method, and it may be suitable for adoption as the new industry standard antibacterial activity quantification method.

## Methods

### Honey samples

A total of 77 honeys were examined in this study. Sixty-nine honeys obtained from Western Australian apiary sites were provided as intentionally blinded sampled by ChemCentre, Bentley, Western Australia as part of its Bee Industry Council of Western Australia (BICWA) research program. An additional eight comparator honeys were examined including one of each of the following; Jarrah from *Eucalyptus marginata* (Jarrah 1), Marri from *Corymbia calophylla* (Marri 1) (both provided by ChemCentre), a multifloral honey with no specific floral source (Capilano, Richlands, Queensland), one Manuka (*Leptospermum*) honey labelled TA 5+ and one labelled TA 10+ (Barnes Naturals, Maryborough, Victoria), Activon Manuka honey (Advancis Medical, UK) and artificial honey prepared as described previously [36]. Levels of methylglyoxal for the Manuka honeys were not stated. An additional Marri honey (Marri 2) was used in some of the validation experiments as it was shown previously in our laboratory to have an “intermediate” level of activity. All honeys were stored protected from light at room temperature (~ 22°C) for the duration of the study. All honeys were stirred thoroughly prior to weighing out portions for each test. For antibacterial activity tests, honey solutions were prepared as weight/volume solutions in sterile distilled water and honey was dissolved completely with the aid of a vortex mixer. Honey solutions were used in assays within 1 h of preparation.

### Microorganisms

Reference strains were obtained from culture collections of The University of Western Australia and PathWest Laboratory Medicine WA. *Staphylococcus aureus* ATCC 25923 was used in the phenol equivalence assay as it is the standard, recommended strain [14]. The four reference strains *Staphylococcus aureus* ATCC 29213, *Enterococcus faecalis* ATCC 29212, *Escherichia coli* ATCC 25922 and *Pseudomonas aeruginosa* ATCC 27853 were used for determining MICs by the broth microdilution assay and for the new antibacterial activity test. These strains were selected as they are the recommended quality control strains for broth antibacterial susceptibility testing according to both the Clinical and Laboratory Standards Institute (CLSI) [37] and the European Committee on Antimicrobial Susceptibility Testing (EUCAST) [38]. All organisms were stored at -80°C in Brain heart infusion broth/glycerol stocks. Working cultures were cultured on blood agar then stored at 4°C, and were refreshed from frozen stocks every four weeks.

### Antibacterial activity measurements

#### Phenol equivalence assay

The PE assay was performed as described previously [10, 12, 14]. An overnight culture was prepared by inoculating 1–2 colonies of *S*. *aureus* ATCC 25923 into approximately 10 ml of Trypticase Soy broth (Becton Dickinson BBL™) and incubating at 37°C with shaking at 125 rpm for 18 h. Following incubation, cells were collected by centrifugation and the cell pellet was then resuspended in sterile 0.85% saline. The density of the cell suspension was adjusted to an absorbance of 0.5 at 540nm. A 100 μl volume of the adjusted inoculum was added to 150 ml of sterile, molten Nutrient agar (Oxoid, Hampshire UK) that had been cooled to approximately 50°C. The agar was swirled gently to mix, then poured into a sterile square bioassay dish measuring 245 mm × 245 mm (ThermoScientific Nunc™ NUN240835). A spirit level was used to ensure that the bench area was level prior to pouring the agar. The agar was left to cool completely at room temperature and was then stored overnight at 4°C in an airtight container. The following day, the agar dish was removed from 4°C storage and allowed to equilibrate to room temperature. A sterile stainless steel cork borer was used to cut 63 equally spaced wells of 8 mm diameter into the agar. Wells were cut 2.5 cm apart in an 8 × 8 grid, leaving one grid space uncut for the placement of an antibiotic control disc. Wells were numbered using a quasi-Latin square to ensure that samples and controls (in duplicate) were placed randomly in the dish. Solutions of honey were prepared at a final concentration of 25% (w/v) in sterile distilled water ensuring complete dissolution with the aid of a vortex mixer. Honey solutions were not routinely filter sterilised prior to testing. If inhibition zones were not readable due to the growth of endemic honey microorganisms, the assay was repeated using a filter sterilised solution of honey. A stock solution of 10% (w/v) phenol (Product #366, Ajax Finechem, ThermoFisher) was prepared in sterile distilled water and from this, further solutions with final concentrations of 2%, 3%, 4%, 5%, 6%, and 7% were prepared in sterile distilled water. Phenol solutions were stored at 4°C and were used for no more than four weeks, after which fresh solutions were prepared. Volumes of 100 μL of each honey solution or phenol standard were pipetted into the allocated wells of the assay dish in duplicate. A trimethoprim antibiotic disc (5 μg; Oxoid, Hampshire UK) was placed in the appropriate position on the assay dish as a positive control. The use of an antibiotic disc is not included in the originally described method, but has been included here as an additional quality control measure. A well with 100 μl of sterile distilled water was included as a solvent control. The assay dish was then incubated for 18 h at 37°C. After incubation, all zones of inhibition were measured by eye and using a ruler to the nearest millimetre. Each zone was measured at least twice in different directions (preferably at right angles) to ensure that well diameter measurements were representative. The mean of the duplicate zone measurements was determined and these values were squared. A linear standard curve was then generated from the mean squared diameter of zone sizes for phenol solutions. The r^2^ value for standard curves was always >0.95. The “phenol equivalence” or “total activity” value for each honey sample was then determined using the equation derived from the phenol standard curve. To correct for both the honey dilution factor of 1 in 4 and the mean density of honey (assumed to be 1.35g/mL), values derived from the standard curve were multiplied by 4.69 [14]. The assay was repeated in entirety on at least two separate occasions and the mean PE value was determined from replicate values.

#### Broth microdilution assay

MICs were determined for all honeys against *S*. *aureus* ATCC 29213, *E*. *faecalis* ATCC 29212, *E*. *coli* ATCC 25922 and *P*. *aeruginosa* ATCC 27853, using the standard broth microdilution method described by the Clinical and Laboratory Standards Institute [31], with minor modifications. Briefly, inocula were prepared by culturing organisms for 18–24 h at 37°C on blood agar, then suspending colonies in 0.85% saline. The density of the cell suspension was adjusted to 0.5 McFarland for *S*. *aureus*, *E*. *coli* and *P*. *aeruginosa* and to 1.0 McFarland for *E*. *faecalis*, all corresponding to approximately 1 to 2 × 10^8^ colony forming units (CFU) per ml. Each standardised suspension was then further diluted in 4 × Mueller Hinton broth for use in the assay. This concentration of Mueller Hinton broth was required to compensate for subsequent dilution with the volume of honey contained in the microtitre plate wells.

Solutions of honey were prepared at 40% (w/v) in sterile distilled water then filter sterilised by passing through a 0.7 μM glass fibre syringe pre-filter to remove larger particles and debris, followed by a 0.2 μm syringe filter to sterilise the solution. Appropriate volumes of each sterile honey solution, ranging from 10 μl to 150 μl were dispensed into wells of a 96-well microtitre plate (Nunc MicroWell NUN260860), and then corresponding volumes of sterile distilled water ranging from 140 μl to 0 μl were added to each well to result in total volumes of 150 μl per well. After the addition of 50 μl volumes of inocula to each well, final inocula concentrations were approximately 5 × 10^5^ CFU/ml, and wells contained final concentrations of honey ranging in 2% increments from 2% to 30% in total well volumes of 200 μl. The positive growth control well contained 150 μl of sterile distilled water and 50 μl of inocula only. Microtitre plates were incubated for 20 h (± 2 h) at 36°C (± 1°C), after which MICs were determined visually as the lowest concentration of honey completely inhibiting growth. MICs were determined for all honeys against all organisms on at least two separate occasions. To enable calculations and statistical analyses, any results that were below the minimum test concentration of 2% honey were given a value of 1%, and any values greater than the maximum test concentration (30%) were given a value of 32%. Where possible, the mode was selected from the two replicate values as the final MIC. If replicate values were not identical, and they differed by ≤4% honey the arithmetic mean was calculated as the final MIC. Where replicate MICs differed by ≥6% honey, the assay was repeated to generate a third value, after which the mode was selected as the final value. If all three values differed, the arithmetic mean was determined as the final MIC.

#### New method for quantification of activity

The new antibacterial activity test was adapted from the CLSI broth microdilution assay [31] and was performed as described for the modified broth microdilution assay above, with minor modifications. Honey solutions, corresponding volumes of sterile distilled water and inocula were dispensed into wells of a 96-well microtitre plate as described above, with the exception that after inoculation the final concentrations of honey in each well were 30%, 25%, 20%, 15%, 10% and 5% (w/v), in final well volumes of 200 μl. The positive growth control well was prepared as described above. The antibiotic tetracycline was tested in parallel on each test occasion as a control. Tetracycline was selected because quality control reference MIC ranges are available for all four organisms [37]. Immediately after inoculation, the optical density of all microtitre plate wells was determined at 600nm using a spectrophotometer microplate reader. These optical density values served as blanks for each well. The plate was then incubated for 20 h (± 2 h) at 36°C (± 1°C) after which the optical density of all microtitre plate wells was determined again at 600nm. This incubation period was selected because preliminary tests indicated that incubation beyond 22 h could result in inconsistent changes in the optical density of some wells containing honey, where some optical density measurements would increase whilst others would decrease. Optical density values determined at time zero were subtracted from values obtained from corresponding wells at 22 h to generate net optical density values. For the antibiotic control, the MIC was determined visually as the lowest concentration resulting in an optically clear well. If the tetracycline MICs were within acceptable quality control ranges this indicated that inoculum density, growth medium, and incubation conditions were optimal for test performance. If the tetracycline MICs were outside acceptable quality control ranges the test was repeated. The entire assay was repeated at least twice per honey.

*Calculation of antibacterial activity values*. The assay generated 24 net optical density values for each honey, obtained from the six different honey concentrations tested against the four different test organisms. Growth relative to the positive control was then calculated by dividing the net optical density value for each honey concentration by the untreated positive growth control for each organism and concentration of honey, and multiplying by 100 to generate a percentage. Any resulting negative values were ascribed a value of zero and any values greater than 100 were ascribed a value of 100. The use of these relative growth values to generate a single measure to represent antibacterial activity was then determined after a series of trial and error calculations. The final approach taken was to assign “antibacterial activity units” to the degree or extent of growth inhibition, whereby more activity units were allocated to higher growth inhibition and fewer activity units were allocated to lower inhibition of growth. The activity units assigned to relative percentage growth values were as follows: relative growth of <10% was assigned 32 activity units; ≥10% to <30% was assigned 16, ≥30% to <50% was assigned 8, ≥50% to <70% was assigned 4, ≥70% to <90% was assigned 2 and ≥90% was assigned 1. The sum of activity units assigned to all 24 conditions (4 organisms × 6 honey concentrations) was then determined, which became the final antibacterial activity value for the honey. A series of variations to this formula was investigated to determine whether any organism or honey concentration could be eliminated to make the assay more efficient without compromising its discriminatory power.

*Assay validation and verification*. Parameters including limit of detection, limit of quantitation, measuring interval, limit of reporting, sensitivity, selectivity and repeatability [39–41] were investigated as described below. Several additional validation parameters such as trueness and linearity of calibration were not relevant to the current method as they relate specifically to the quantification of levels of a particular analyte or compound present within a matrix, rather than biological activity. Sources of variation within the assay were initially identified and optimised as part of the protocol optimisation and pre-validation, some of which are described below under robustness. Unless stated otherwise, the antibacterial activity protocol was performed exactly as described above for each of the validation studies below.

*Limit of detection*. The limit of detection is defined as the mean of the lowest detectable value or smallest detectable concentration of an analyte, plus three times the standard deviation. Since limit of detection testing is usually applied to a specific, detectable analyte, rather than antibacterial activity, it is not straightforward as to which substance should be used to indicate the limit of detection of antibacterial activity. Therefore, a substance with the lowest possible level of antibacterial activity (in fact, no detectable activity under the test conditions), sterile distilled water, was used as a surrogate substance. The sterile distilled water was used in place of honey in the antibacterial activity assay, while all other aspects of the method were unchanged. The final antibacterial activity value was generated for nine replicates, and the mean and standard deviation was calculated.

*Limit of quantitation (instrumental)*. The instrumental limit of quantitation was investigated to determine the extent to which the microplate reader could discriminate between different optical densities and by extension, differences in bacterial growth. This was determined by diluting overnight broth cultures of each test organism in sterile distilled water and dispensing into a 96 well plate at different concentrations to create a known concentration gradient. The highest concentration was 100% broth culture and each successive dilution decreased by 10% to a final concentration of 0% broth culture, which consisted of sterile distilled water only. Four replicate wells were used per culture concentration per organism. The optical density of all wells was measured immediately using a microplate reader at 600nm. After subtraction of the optical density measurements for blanks, the concentration of culture relative to the 100% broth culture was calculated for each replicate, and a mean was generated.

*Measuring interval*. The maximum value was determined by calculating the theoretical antibacterial activity value that would be derived if growth was inhibited by ≥90% in all 24 wells containing honey, and the optical density was <10% relative to the control. The minimum of the measuring interval is defined as greater than, or equal to the limit of detection.

*Sensitivity*. The capacity for the assay to differentiate between two very similar samples was investigated by comparing two groups of honeys with very similar, but not identical activity as defined by MICs. Group A consisted of seven different honeys and group B consisted of nine different honeys. Antibacterial activity values for the two groups were analysed using Student’s t-test (two-tailed, assuming equal variance; P = 0.05).

*Repeatability*. Repeatability tests were conducted to determine whether the assay produced similar results when all experimental and technical factors were identical. Repeatability was investigated by testing a single honey sample eight times at the same time by the same operator. Three different honey samples were investigated to cover a range of antibacterial activity and to determine repeatability across the entire measuring range. Jarrah 1 was used to assess the high activity range, “artificial honey” was used to assess the low activity range, and Marri 2 was used as an intermediate level honey. Results were analysed by calculating the coefficient of variation (CV) for each stage of the assay and for the final antibacterial activity values.

*Reproducibility*. Inter-operator variability was investigated by having two operators perform the test in different laboratories with different equipment. Each operator tested Jarrah 1, Marri 2 and artificial honeys three times each. Results for each operator and honey were analysed by Student’s t-test (two-tailed, assuming equal variance; P = 0.05).

*Ruggedness*. Unless stated otherwise, all ruggedness studies were performed using Jarrah 1, Marri 2 and artificial honeys, and results obtained using variant assay conditions were then compared to results obtained using standard assay conditions. The effect of inoculum growth phase was investigated by comparing standard inocula preparation (direct suspension from an overnight culture on Blood agar) to exponential phase inocula prepared by growing organisms in Trypticase Soy broth for approximately 3 h at 37°C with shaking. The effect of inoculum density was investigated by conducting the assay with a light inoculum (1 log less dense than standard inoculum) and a heavy inoculum (1 log more dense than standard inoculum). The effect of assay incubation time was investigated by using standard assay conditions, then determining the optical density of each well at 600nm at 18, 20, 22 and 24 h. The effect of time elapsed between preparing the honey solution by dissolving in sterile distilled water, and inoculating the test with bacteria was also investigated. This was examined because it is known that the addition of water to honey enables the enzyme glucose oxidase to catalyse the reaction that produces hydrogen peroxide, which is an antibacterial component of honey. To examine this, solutions of Jarrah 1 and Marri 1 were prepared at 40% (w/v) in sterile distilled water and incubated at room temperature (~22°C) for 24 h. Aliquots were removed after 1, 2, 4 and 24 h and tested using standard antibacterial activity assay conditions. Evaluation of whether different microplate readers would produce different results was investigated by obtaining optical density measurements for the same assay in a 96-well plate on two different instruments, which were the SpectraMax® 190 Microplate reader (Molecular Devices) and the xMark™ Microplate Absorbance Spectrophotometer (Bio-Rad), then comparing optical density values. Similarly, plate manufacturer was investigated by conducting the standard assay in four different types of 96-well microtitre plates (NUN260860, NUN167008, Falcon and CellStar). Antibacterial activity values obtained in the different plates were analysed by one-way ANOVA (P = 0.05).

#### Data analyses

Unless stated otherwise, each assay was repeated at least twice on separate occasions and mean values determined. The mean MIC for each honey was determined as the arithmetic mean of the four MICs obtained for each organism, for the purposes of comparing antibacterial activity measures. The relationship between values obtained using each of the three antibacterial activity tests was analysed using Pearson’s correlation (two-tailed, level of significance 0.05).

## Results

### Phenol equivalence values

**Fig 1:**
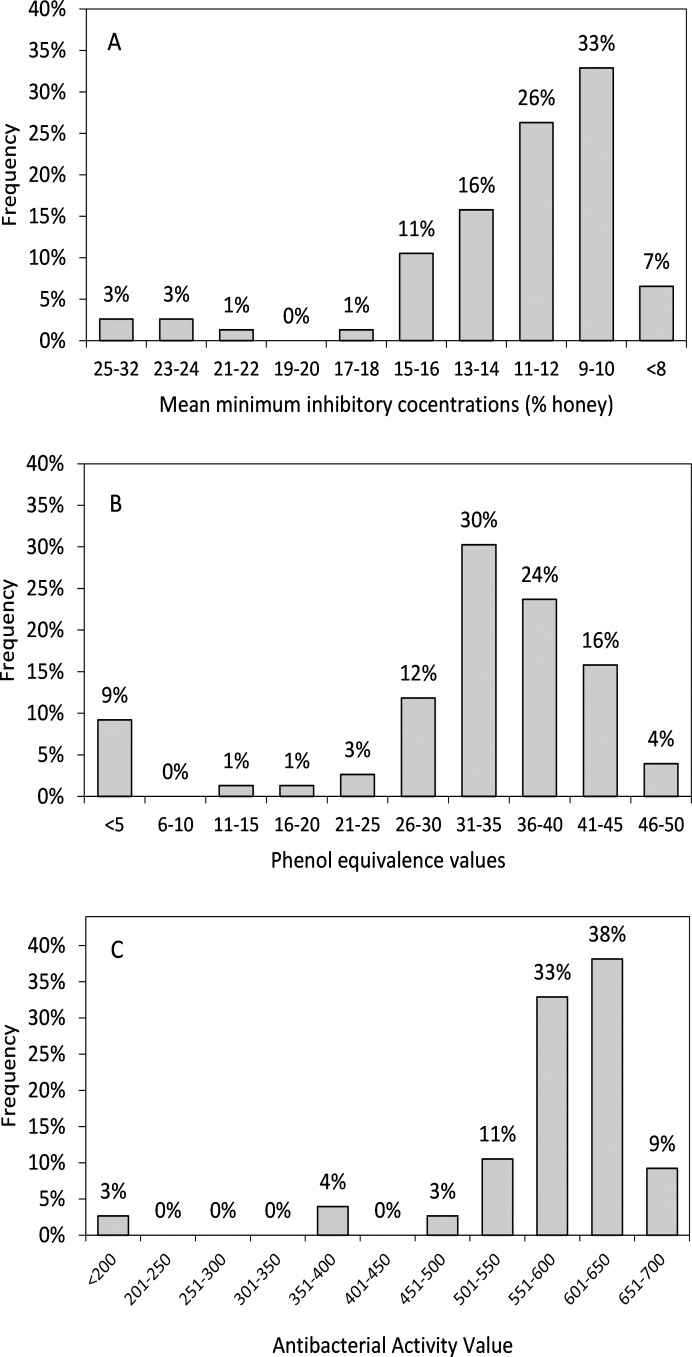
Distribution of antibacterial activity values obtained for 77 honeys using three testing methods. (A) Mean minimum inhibitory concentrations (% w/v) obtained by broth microdilution using *Staphylococcus aureus* ATCC 29213, *Enterococcus faecalis* ATCC 29212, *Escherichia coli* ATCC 25922 and *Pseudomonas aeruginosa* ATCC 27853. (B) PE values determined using *Staphylococcus aureus* ATCC 25923. (C) New assay activity units, determined from relative growth of *S*. *aureus* ATCC 29213, *E*. *faecalis* ATCC 29212, *E*. *coli* ATCC 25922 and *P*. *aeruginosa* ATCC 27853 cultured with six different concentrations of honey. Each measurand was grouped into categories for the purpose of plotting the histograms.

PE values for the 69 blinded samples ranged from 0 to 48 (% w/v phenol), derived from zones of inhibition ranging from 0 to 22 mm. No zones of inhibition were observed for seven honeys, including the artificial and multifloral honeys, and three (4.3%) of the 69 blinded samples. For the three *Leptospermum* honeys the PE values were zero for both the Activon and the *Leptospermum* TA 5+ samples, and was 11 for the *Leptospermum* TA 10+. PE values for the remaining comparator honeys were 36 for Jarrah 1 and 27 for Marri 1. Marri 2 was not tested using this assay. The distribution of PE values obtained is shown in Fig 1, and shows that over half of the samples (54%) had PE values ranging from 31–40.

**Fig 2:**
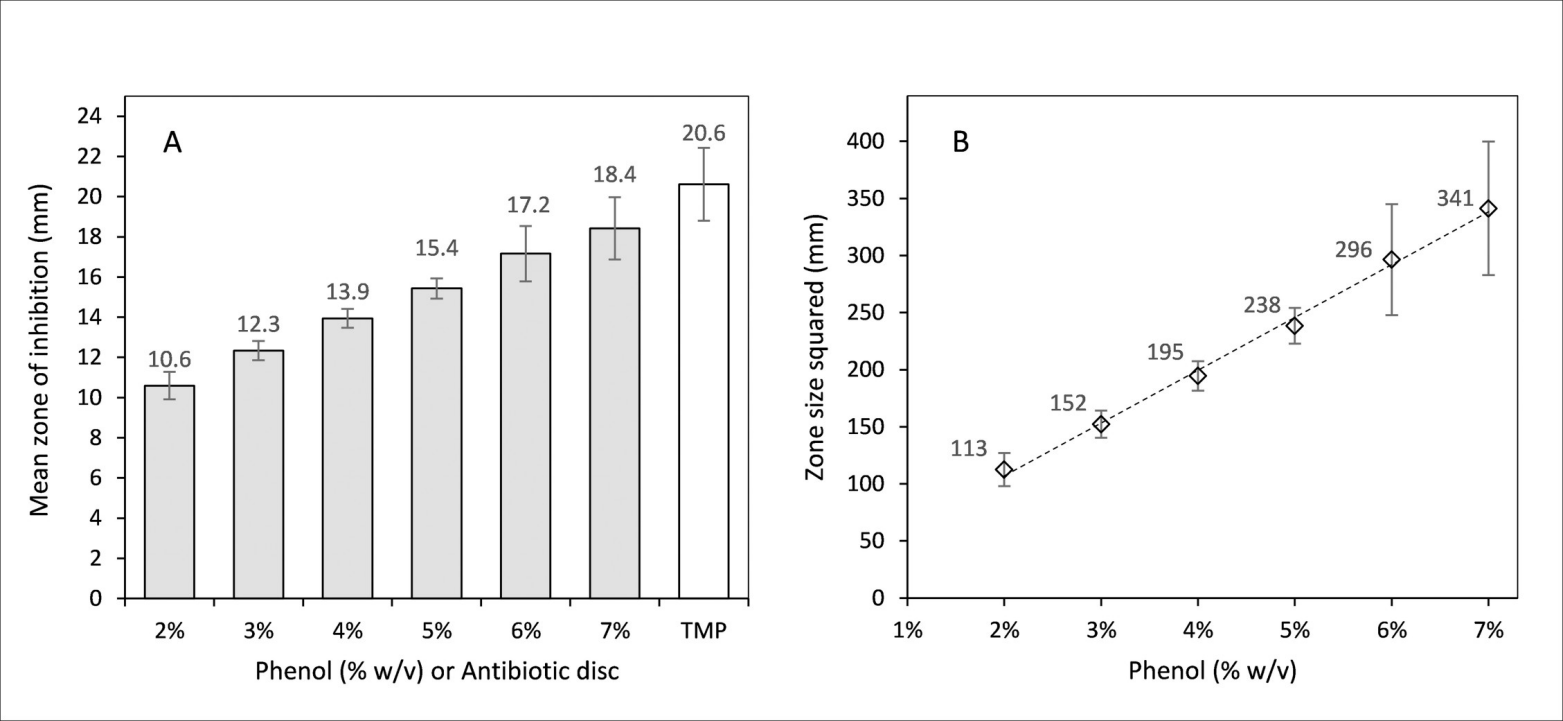
Mean zone sizes for phenol standards and antibiotic disc (A) and phenol standard curve (B) plotted from mean phenol zone sizes. Bars represent standard deviation.

Zones of inhibition obtained for trimethoprim (5 μg disc) ranged from 19–24 mm, with a mean of 21 mm, standard deviation of 1.8 and %RSD of 8.8 (Fig 2). The mean and standard deviation of zones of inhibition obtained for phenol solutions from all experimental repeats, and the average phenol standard curve is shown in Fig 2. The %RSD values for the phenol standards were 6.5, 3.9, 3.4, 3.3, 8.0 and 8.4 for the 2, 3, 4, 5, 6 and 7% phenol solutions, respectively. The limit of detection of antibacterial activity for the PE assay, based on the smallest zone size theoretically measurable of 9 mm for a solution of 25% honey, and using the standard curve shown in Fig 2B, is 6.8 (% w/v phenol).

### Minimum inhibitory concentrations

**Fig 3:**
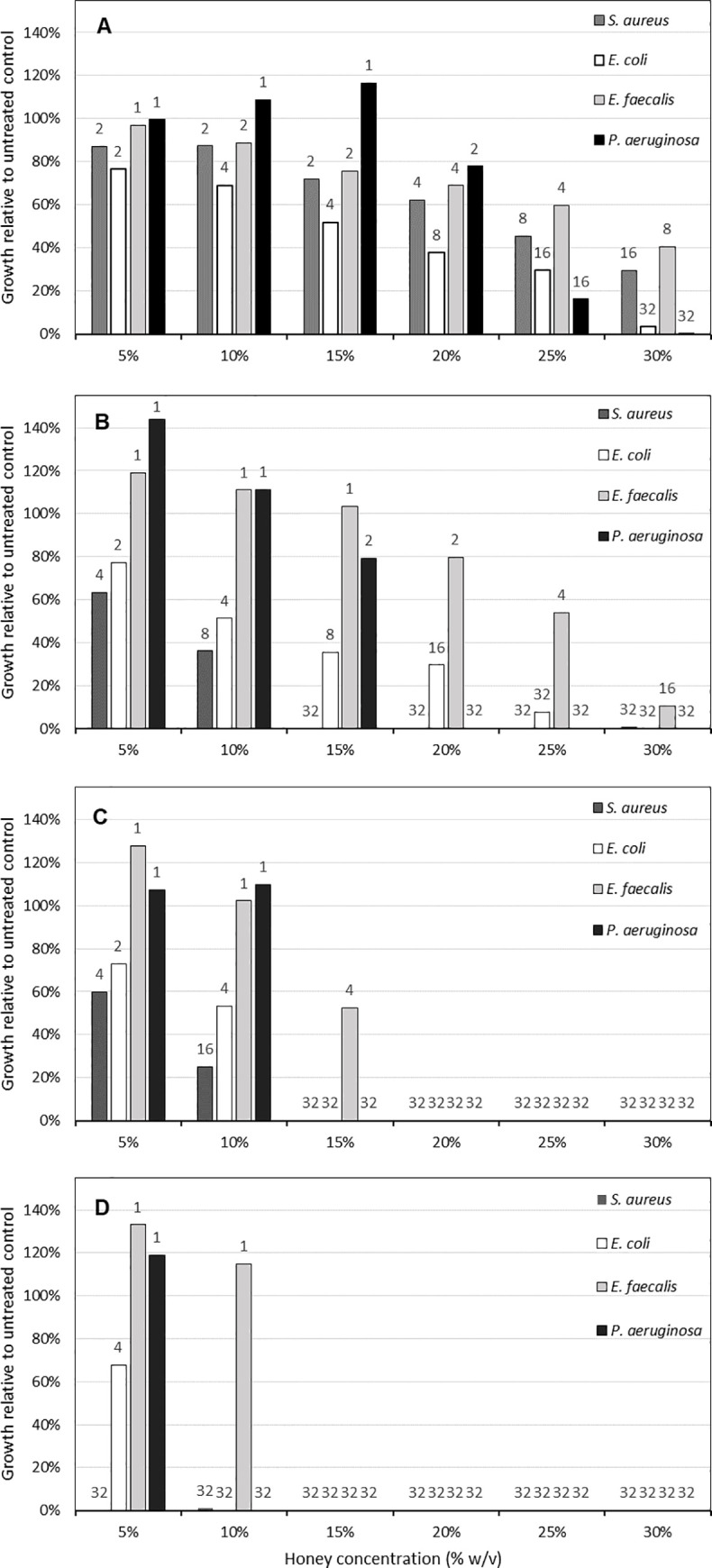
Representative honeys showing varying levels of antibacterial activity. Bars show the growth of test organisms relative to the positive growth control. Numbers above bars show the values ascribed to the level of growth inhibition, which when added together give the new antibacterial activity value. (A) Relatively low activity honey (174 activity units). (B) Moderately low activity honey (359 activity units). (C) Moderate activity honey (514 activity units). (D) Relatively high activity honey (647 activity units).

The mean of the MICs obtained for all four organisms for each honey was determined to enable comparison of overall activity between honeys. For the 69 blinded samples, mean MICs ranged from 6.5 to 23.5 (% w/v honey) and the distribution of mean MIC values is shown in Fig 1B. MICs for *S*. *aureus* ATCC 29213 across all 69 blinded honey samples ranged from <2% to 25%, with a mean of 5% and a mode of 4% (Fig 3). Four of the honeys showed MICs of less than 2% against *S*. *aureus* and a value of 1% was ascribed to these for the purposes of data analyses. For *E*. *faecalis* ATCC 29212, MICs for the 69 honeys ranged from 10% to >30%, with a mean of 17% and a mode of 14%. For *E*. *coli* ATCC 25922, MICs ranged from 8% to 27%, with a mean of 12% and a mode of 10%. Lastly, MICs for *P*. *aeruginosa* ATCC 27853 ranged from 6% to 20%, with a mean of 10% and a mode of 8%. Overall, MICs were the lowest for *S*. *aureus*. MICs were generally highest for *E*. *faecalis*, whereas *E*. *coli* and *P*. *aeruginosa* were largely similar. MICs for artificial honey were >30% for all organisms except *P*. *aeruginosa* for which the MIC was 28%, and for multifloral honey the MICs were >30% for both Gram positive organisms and were 29% for *E*. *coli* and 26% for *P*. *aeruginosa*. As a generalisation, all honeys showed the same broad trends in antibacterial activity across the four test organisms, whereby *S*. *aureus* was always the most susceptible, followed in order by *P*. *aeruginosa*, *E*. *coli* and *E*. *faecalis*.

### New method for quantification of antibacterial activity

#### Optical density measurements

Bacterial cell density was quantified by determining the optical density of all cultures at 600nm. Analysis of optical density data obtained for all experimental repeats (n = 154; obtained from two experimental repeats for 77 test honeys) showed the following mean (± standard deviation) OD values for the positive growth control wells: *S*. *aureus* 0.57 (± 0.17); *E*. *coli* 0.81 (± 0.09); *E*. *faecalis* 0.34 (± 0.15) and *P*. *aeruginosa* 1.02 (± 0.11). The organism showing the most variation in optical density measurements for the positive growth control was *E*. *faecalis*, followed by *S*. *aureus*. Optical density measurements obtained after growth in the presence of each honey concentration varied considerably, depending on the concentration of honey tested, the level of activity of each honey, and the intrinsic susceptibility of each test organism. As a generalisation, all four test organisms were capable of growth at almost all honey concentrations for those honeys with relatively low activity (Fig 3A). For honeys with relatively high activity, test bacteria were only able to grow in the presence of 5% and occasionally 10% honey, with the exception of *S*. *aureus* which was generally not able to grow in these conditions (Fig 3).

**Fig 4:**
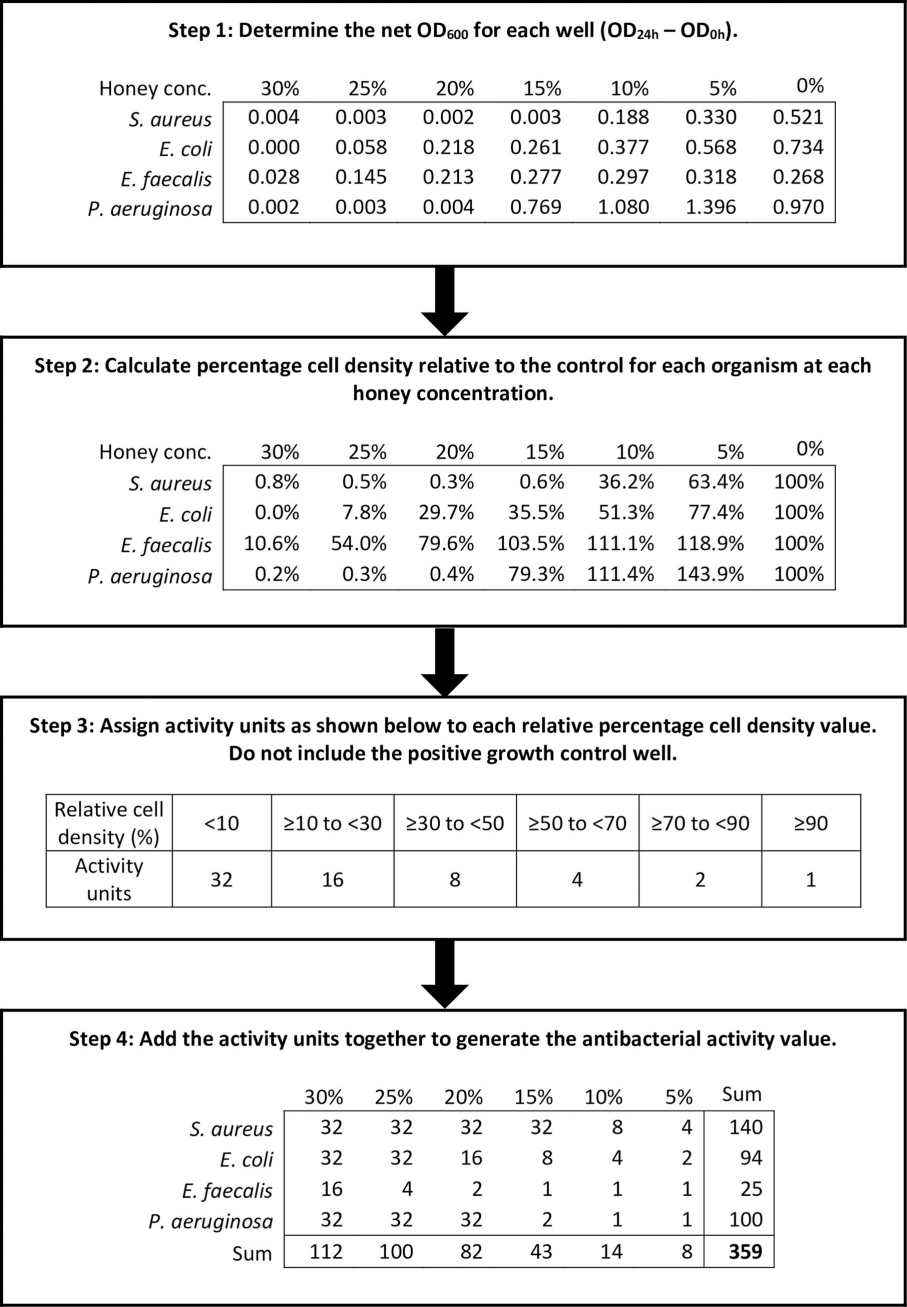
Representative example of the calculation required for the new antibacterial activity value. Step 1 is to determine the net optical density values; step 2 is to calculate the percentage growth relative to the positive control; step 3 is to assign the activity units to each relative percentage growth value and the final step is to add the 24 values together. Data for this honey is also illustrated in Fig 3B.

The percentage growth relative to the untreated positive growth control was determined for each organism at each concentration of honey. These relative growth percentages were used to calculate the new antibacterial activity value as described below and shown in Figs 3 and 4. In addition, and to provide an overview of broad trends in bacterial growth inhibition, the average of these relative growth percentages was determined for each organism and for all honeys excluding the artificial and multifloral honeys as these two honeys only moderately inhibited bacterial growth. Comparison of test organisms showed that *S*. *aureus* was the most susceptible to honey, producing essentially no growth in the presence of 25% or 30% honey (Table 1), with the exceptions of the artificial and multifloral honeys. Similarly, growth was largely absent in the presence of 15 and 20% honeys. The mean relative growth of *S*. *aureus* was <2% in the presence of ≥15% honey (Table 1). Mean relative growth of *S*. *aureus* in the presence of 5% honey was 20.2%, and was 6.7% in the presence of 10% honey. In contrast, the least susceptible organism was *E*. *faecalis*, with mean relative growth of 0.76%, 4.23%, 11.70%, 50.61%, 104.85% and 127.28% in the presence of 5, 10, 15, 20, 25 and 30% honey, respectively. Examination of standard deviation values showed that the highest variation occurred at 5% for *S*. *aureus*, 10% for *E*. *coli* and *P*. *aeruginosa* and 15% for *E*. *faecalis*, indicating that these are the most highly discriminatory concentrations for each test organism. Stimulation of growth occurred at 5% honey, and most commonly occurred in *P*. *aeruginosa*, with growth stimulated (>100% relative to the untreated control) in 147 of the 154 (95%) tests conducted. Similarly, *E*. *faecalis* growth was also stimulated at 5%, occurring in 128 of the 154 (83%) tests. Growth stimulation also occurred at 10% honey although to a much lesser extent, and occasionally also occurred at 15% honey.

**Table 1:** Relative percentage growth of each organism at each concentration of honey, for all honeys excluding artificial and pasture.

Honey concentration		Mean relative percentage growth			
(w/v)		*S*. *aureus*	*E*. *coli*	*E*. *faecalis*	*P*. *aeruginosa*
		ATCC 29213	ATCC 10418	ATCC 29213	ATCC 27853
5%	Mean (± S.D.)	20.2 ± 41.0	66.6 ± 15.8	127.3 ± 37.1	134.4 ± 24.8
	Range	-2.9–221.3	0.0–131.9	0.0–245.3	45.1–208.2
10%	Mean (± S.D.)	6.7 ± 27.1	28.5 ± 23.8	104.8 ± 39.8	33.8 ± 55.3
	Range	-2.0–261.2	0.0–93.5	0.0–280.5	0.0–201.5
15%	Mean (± S.D.)	1.9 ± 12.4	5.1 ± 13.6	50.6 ± 49.4	5.6 ± 20.6
	Range	-1.9–124.8	0.0–78.8	0.0–228.6	0.0 = 116.5
20%	Mean (± S.D.)	0.7 ± 4.4	1.6 ± 7.1	11.7 ± 26.2	0.4 ± 2.7
	Range	-0.6–43.2	0.0–58.6	0.0–122.6	-0.2–78.0
25%	Mean (± S.D.)	0.2 ± 1.4	0.4 ± 2.2	4.2 ± 14.1	0.0 ± 0.1
	Range	-1.2–15.8	0.0–47.9	0.0–71.8	0.0–16.4
30%	Mean (± S.D.)	0.0 ± 0.3	0.1 ± 0.3	0.8 ± 3.6	0.0 ± 0.1
	Range	-2.6–1.3	0.0–15.4	0.0–83.4	0.0–0.9

#### Development of the calculation for the antibacterial activity value

Each honey test generated 24 relative growth values (6 honey concentrations × 4 organisms) which were then used to generate a single antibacterial activity value, as shown in Fig 4. Using the new assay, values obtained using the calculation formula for the 69 blinded honey samples ranged from 368 to 669 activity units. The mean and standard deviation was 596 and 57, respectively, and the distribution of values is shown in Fig 1. The majority of honeys (71%) had values in the range of 551–600 activity units, with relatively few (9%) having values higher than this. Values for comparator honeys were 181 for artificial honey, 148 for multifloral honey, 372 for *Leptospermum* NPA 5+, 538 for *Leptospermum* NPA 10+, 517 for Activon honey, 572 for Jarrah 1 and 511 for Marri 1. Representative examples of relative growth in the presence of several honeys, with corresponding antibacterial activity values are shown in Fig 3.

A number of variations to the calculation were investigated to determine whether a similar level of discrimination between levels of antibacterial activity could still be achieved by using fewer organisms or fewer concentrations of honey. If so, the method could potentially be simplified thereby saving on resources, time or both. However, results showed that removal of any one parameter was detrimental to the final antibacterial activity values as the change typically led to a loss of power to be able to discriminate between different levels of antibacterial activity. As such, all experimental parameters were retained. Several examples are described below to illustrate the effect of removing one or more parameter. For example, it was considered that results for *E*. *coli* may not be critical in the calculation given that the pattern of growth inhibition was somewhat similar to that of *P*. *aeruginosa*. However, removal of *E*. *coli* values resulted in loss of discrimination, meaning that all antibacterial activity values were then clustered closely together and activity between honeys could not be distinguished. A similar result occurred when *P*. *aeruginosa* values were removed from the calculation instead of *E*. *coli*. Next, since *E*. *faecalis* showed the least amount of growth inhibition by honey (especially when compared to *S*. *aureus*) it was considered that the *E*. *faecalis* values may not make a useful contribution to the assay. However, removal of *E*. *faecalis* values also resulted in a loss of discrimination and this also highlighted that *E*. *faecalis* is actually a very discriminatory organism and provides a wide spread of useful information for the assay. Similarly, because *S*. *aureus* can be inhibited at particularly low concentrations, it is an ideal organism for demonstrating very high antibacterial activity in this assay. Lastly, it was common for there to be no bacterial growth in wells containing 30% and 25% honey. As such, it was conceivable that these concentrations may not make a useful contribution to calculating final antibacterial activity value. However, results showed that these concentrations were important for discriminating between honeys with moderate and low activity. Since the assay must be able to quantify activity for a broad range of honeys, all organisms and all six honey concentrations were retained in the final calculation formula.

#### Assay validation

Results of method validation are described below and shown in Table 2. The limit of detection for the new assay was determined to be 53 activity units (Table 2). This value was generated by determining the mean antibacterial activity value for sterile distilled water (no antibacterial activity), which was 37.9 with a standard deviation of 4.95. Therefore, the calculated limit of detection for this assay was 37.9 + (3 × 4.95), which is equal to 52.75.

**Table 2:** Results of method validation.

Validation Parameter	Result
**ACCURACY**	
Limit of Detection	53 activity units
Limit of Quantitation (instrumental)	The instrument can quantify a difference of at least 10% in bacterial cell density. Optical densities of dilutions of bacterial cultures showed a linear trend
Sensitivity	Two groups of honeys with very similar activity shown to be significantly different (P = 0.0499)
Selectivity	The use of blanks corrects for any interference within the assay and ensures that only bacterial optical density is measured
**PRECISION**	
Repeatability (% RSD)	% RSD calculated for the final antibacterial activity values were 1.8% (Jarrah 1) 3.1% (Marri 2) and 6.7% (Artificial)
Reproducibility (inter-laboratory)	Minor inter-operator variability was evident but results did not differ significantly. P values were 0.479 (Jarrah 1), 0.138 (Marri 2) and 0.183 (Artificial)
**RUGGEDNESS/ROBUSTNESS**	
Instrument variability	Minimal variation whereby two different instruments produced similar results
Inoculum growth phase	Results produced using stationary and exponential phase inocula differed significantly for *P*. *aeruginosa* (P = 0.0058) but not for the other organisms
Assay incubation time	Optical density values were stable at 18, 20 and 22 h, but unstable thereafter
Honey solution time elapsed	Activity of honey solutions increased over time; time between preparing honey and inoculation recommended as ≤ 1 h
Microtitre plate type	Results obtained in different microtitre plates differed significantly for Artificial honey only. P values were 0.1415 (Jarrah 1), 0.389 (Marri 2) and 0.0356 (Artificial)
**OTHER**	
Measuring Interval	53–768 activity units
Matrix Effects	Not applicable: relevant to the quantification of a specific analyte within a matrix, rather than activity
Trueness/Bias	Not applicable: not investigated due to the lack of standard reference material
Linearity	Not applicable: not investigated due to the lack of standard reference material
Measurement Uncertainty	Not within the scope of this study

Limit of instrument quantitation studies showed that optical density values measured by the spectrophotometric microplate reader were higher than, or equal to, the calculated theoretical cell density concentrations by an average of 1.32% to 8.63%, and followed a linear trend (S1 Appendix). Results showed that the instrument is capable of determining a difference of at least 10% between known concentrations of bacterial culture, which is a sufficient level of discrimination for the assay.

The measuring interval was determined to be 768 to 53 activity units. The maximum antibacterial activity value of 768 was determined based on a hypothetical honey that resulted in complete inhibition of bacterial growth at every concentration of honey and for every organism. This value was obtained by assigning all 24 microplate wells the maximum value of 32 activity units, indicating maximum growth inhibition, and adding these to generate the value of 768. Any honey samples that exceed this value cannot be differentiated, so this is therefore determined as the maximum of the measuring interval. The minimum of the measuring interval is defined as greater than or equal to the limit of detection, which was determined as described previously to be 52.75 activity units. However, the theoretical minimum value for the assay is 24, obtained by assigning 1 activity unit (corresponding to 100% growth relative to the control, representing 0% growth inhibition) to each of the 24 relative growth values.

Several ruggedness parameters were investigated, with varying results. Comparison of inoculum growth phase on growth inhibition by honey demonstrated no significant differences in antibacterial activity values for *S*. *aureus*, *E*. *faecalis* and *E*. *coli*, whereas differences for *P*. *aeruginosa* were significant (P < 0.05), with the exponential phase inoculum being less susceptible to honey. For example, for *P*. *aeruginosa* the net optical density after 24 incubation with 10% (w/v) Marri 1 was 1.34 for the exponential phase inoculum, compared to 0.75 for the stationary phase inoculum. Comparison of different inoculum densities showed that the less dense (light) inoculum did not significantly alter the assay result when compared to standard inoculum density, whereas the more dense (heavy) inoculum resulted in substantial differences in growth (determined by optical density) at one or more concentrations of honey. Assessment of variations in incubation period showed that optical density measurements at 18, 20 and 22 h remained relatively constant, whereas optical density measurements obtained at 24 h either increased or decreased substantially compared to previous time points (S1 Appendix). The optimal incubation period was therefore determined to be 20 ± 2 h. Comparison of optical density measurements for a single 96-well plate obtained on two different spectrophotometric instruments showed almost identical measurements, indicating that the instrument did not represent a significant source of variation. Comparison of the effect of time elapsed between preparing the solution of honey and testing for antibacterial activity showed little effect for Marri 1 but for Jarrah 1 antibacterial activity was increased at 2, 4 and 24 h compared to 1 h, for one or more organism. Activity at 4 h was particularly high (S1 Appendix). Based on this, it was concluded that honey solutions should be dispensed into microtitre plates and inoculated within 1 h of preparation to minimise the potential for hydrogen peroxide to accumulate and unduly influence the assay outcome. We have shown in previous research that honeys may differ in both their capacity to generate hydrogen peroxide, and rate of hydrogen peroxide accumulation [42], which may impact results of antibacterial activity assays.

#### Relationship between activity measurements

**Fig 5:**
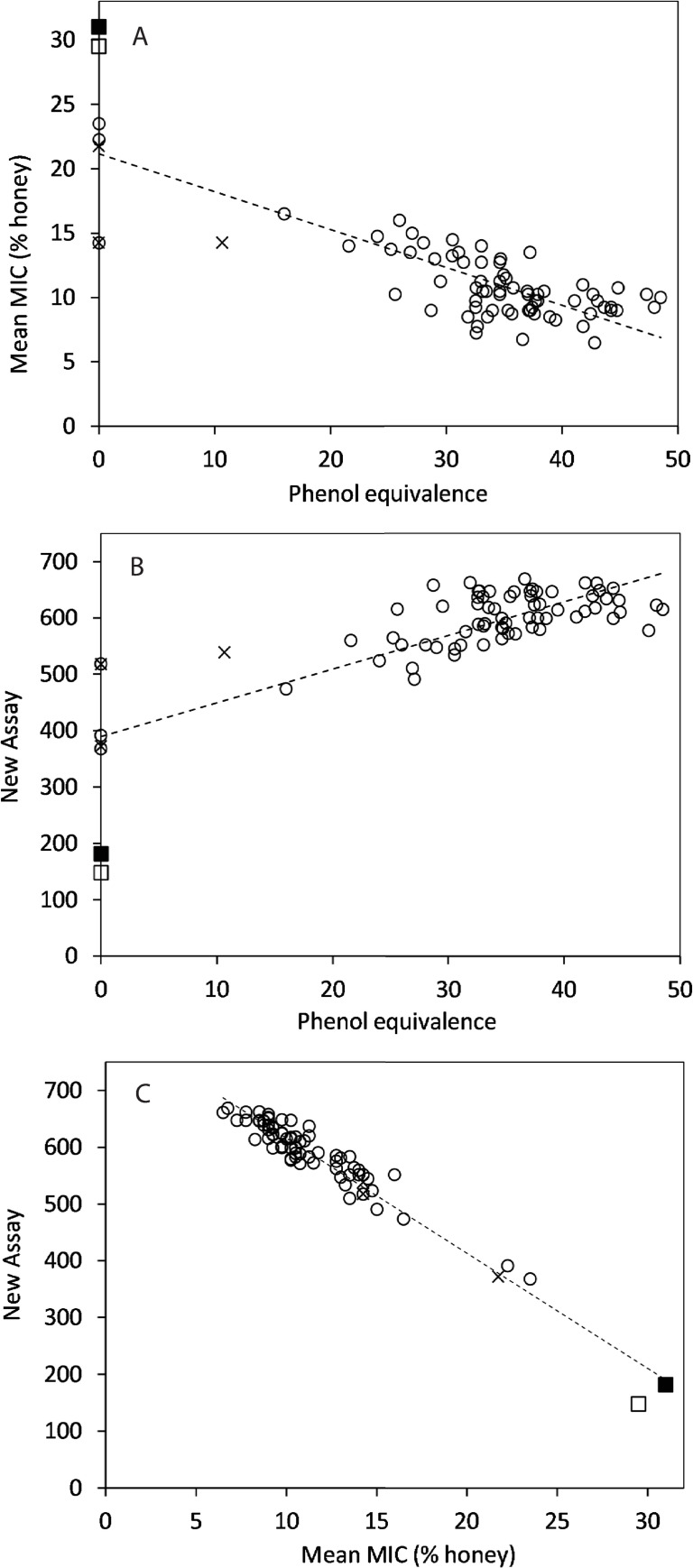
Correlation of antibacterial activity measurements. Scatter plots show correlation between (A) Phenol equivalence and mean MIC values; R^2^ = 0.649, (B) Phenol equivalence and new antibacterial activity values; R^2^ = 0.620 and (C) mean MIC and new antibacterial activity values; R^2^ = 0.949. The open circles represent blinded samples and Jarrah and Marri controls, cross symbol denotes *Leptospermum* honeys, open square represents the multifloral honey and the black square represents artificial honey.

Analysis of antibacterial activity measures obtained for all 77 honeys (69 blinded and eight comparators) by Pearson correlation (2-tailed) showed correlation between values (Fig 5). The weakest correlation was between PE values and the new antibacterial activity values (r = 0.787, p < 0.0001). There was a stronger relationship between PE values and mean MIC values (r = -0.806, p < 0.0001), whereas the strongest relationship was between new antibacterial activity values and mean MIC values (r = -0.974, p < 0.0001). Using the equation from the trend line shown in Fig 5B, new antibacterial activity values can be extrapolated for existing PE values. For example, a PE value of 10 corresponds to an antibacterial activity value of 449, a PE of 20 corresponds to 509, a PE of 30 corresponds to 569, a PE of 40 corresponds to 628 and a PE of 50 corresponds to 688.

Examination of results obtained for individual honeys demonstrated that in general, levels of activity quantified by all three methods were in agreement. That is, if results of one assay indicated relatively high activity, this was likely to be seen across all three assays and likewise if a honey showed relatively low activity by one method, this was likely to be reflected across the remaining two assays. Two notable exceptions were the Activon Manuka honey, which gave a PE value of zero and an antibacterial activity value of 517, and one of the blinded samples which also gave a PE value of zero and an antibacterial activity value of 518.

To further evaluate the extent of agreement between values obtained by the three methods, honey activity measures were ranked from high to low in numerical order. The 25 honeys with highest, or lowest activity were then examined to see how rankings compared across all three assays. For the 25 honeys with the highest activity according to the new assay, 22 (88%) of these honeys were also ranked within the top 25 determined by mean MIC, whereas only 10 (40%) of these honeys were ranked in the top 25 according to PE values. Examination of the lowest 25 values according to new assay showed that 21 (84%) honeys were also ranked in the lowest 25 according to both mean MIC values and PE values. This indicates that methods are broadly in agreement for lower activity honeys, but that in the higher activity honeys, PE and broth results are not necessarily in agreement. When honeys were sorted based on antibacterial activity value and then grouped, the mean antibacterial activity value and mean PE value for each grouping could be determined and compared (Table 3). This provides an approximate indication of how values obtained by the new assay correspond with PE values. Median values were also determined for each activity range but were very similar to mean values and are therefore not shown. Comparison of the relationship between actual mean values shown in Table 3 with theoretical antibacterial activity values determined from the scatter plot shown in Fig 5B shows some similarities. For example, as shown in Table 3, a mean PE of 30.1 corresponds to a mean antibacterial activity value of 560, and from the scatter plot a PE of 30 corresponds to a theoretical antibacterial activity value of 569.

**Table 3:** Comparison of values from the new antibacterial activity scale to the existing phenol equivalence scale. Numbers are the mean ± standard deviation for all values within each specified range.

Range of		Mean ± Standard deviation		
Antibacterial Activity Values	n *	Antibacterial activity value	Phenol equivalence	Mean MIC
>650	7	659.5 ± 6.2	37.6 ± 5.8	8.1 ± 1.1
625–649	16	641.0 ± 7.1	37.0 ± 4.4	9.0 ± 0.9
600–624	14	615.0 ± 7.3	38.7 ± 6.6	10.0 ± 0.8
575–599	15	587.6 ± 8.3	36.4 ± 4.3	11.1 ± 1.3
550–574	9	559.9 ± 8.5	30.1 ± 5.1	13.4 ± 1.6
525–549	4	541.3 ± 6.1	25.2 ± 9.7	13.8 ± 0.7
500–524	4	517.4 ± 5.3	12.7 ± 14.8**	14.2 ± 0.5
400–499	2	482.3 ± 12.4	21.5 ± 7.8	15.8 ± 1.1
300–399	3	377.2 ± 12.6	0.0 ± 0.0	22.5 ± 0.9
<300	2	164.8 ± 23.7	0.0 ± 0.0	30.3 ± 1.1

* A total of 76 honeys are included. Marri 2 is not included.

** Values for two honeys were zero. Exclusion of the two zero values results in a mean of 25.5.

## Discussion

The PE assay (or UMF assay) is currently used by the honey industry to quantify the antibacterial activity of honey. As mentioned in the introduction, it is questionable whether this assay is well suited to measuring the activity of honey, given that it relies on the diffusion of compounds with varying polarities and molecular weights through agar. In addition, variability of results of the PE assay between laboratories has been identified as an issue [11]. Difficulties interpreting zone sizes, particularly for honeys producing zones with an indistinct or hazy edge, or with a halo of faint growth within the zone [11] have also been noted. For agar diffusion assays with antibiotics, reading zone sizes is said to be the hardest aspect of the assay to standardise [43], indicating that this problem is widespread.

Compounding these issues is the lack of publically available validation studies for the PE assay, including intra- and inter-laboratory trials, and quality control reference data for the phenol zone sizes. Quality control data are a critical aspect of any type of quantitative or analytical laboratory testing. In particular, accurate phenol zone sizes are vital as minor changes will alter the phenol standard curve, which in turn affects the final PE value ascribed to the honey. Phenol calibration curves have been published in several previous research papers [14, 44, 45], however, each of these curves differs substantially from the others, and also from the calibration curve in the current study. Variability in phenol zone sizes was observed in the current study, with %RSD values for phenol standards ranging from 3.3 to 8.4. These values are higher than those typically found for chemical analysis methods (1–3% RSD) [46], however, microbial tests are known to have comparatively higher %RSD values [46]. Also, minor differences in zone sizes obtained by disc or agar diffusion are to be expected and are deemed acceptable if they fall within a pre-defined range. Ironically, phenol is not an ideal substance for use in agar diffusion assays. It is relatively non-polar and has limited solubility in water of approximately 8.3 g/100ml [47], which is likely to limit its diffusion through agar. The inclusion of additional assay controls, such as antibiotic discs, can potentially assist in assay validation. In the current study, zone sizes generated for trimethoprim were within the acceptable range of 19 – 26mm specified by CLSI (for the control strain *S*. *aureus* ATCC 25923) [37], but it should be noted that the methodology for the PE assay and the standard antibiotic disc diffusion test differ considerably, meaning that results are not directly comparable.

Relatively few publications describe antibacterial activity results obtained for honeys using the PE assay, which limits the comparison of current data to previous results. Less than 20 publications were identified that utilise the method, and of these, many report a modification to assay parameters, further limiting the direct comparability of results. Modifications include using a test microorganism other than *S*. *aureus* [48] or an alternative strain of *S*. *aureus* [49], a medium other than Nutrient agar [48], or a different size of bioassay plate [32, 45]. Studies using PE methodology the same as, or very close to standard methodology used in the current research have reported total activity measurements (expressed as percent PE) for various honeys (excluding honeys with undetected activity) ranging from 4.2 to 18.8 [49], 12.0–21.2 [12], 7.4–34.3 [10], 7.8–42.6 [50] and ~5–58 [14]. Values from the current study are within the ranges of those reported previously, however, and as alluded to above, the lack of phenol standard curve information in previous publications limits meaningful comparisons.

Given the limitations of agar diffusion-based methods for quantifying the antibacterial activity of honey, a liquid or broth-based assay was considered more appropriate. This bypasses the issue of diffusion through agar, and also provides a direct measurement of activity, rather than activity relative to phenol. The broth microdilution assay was originally developed specifically for assessing the in vitro susceptibility of clinical isolates to antibiotics, with the aim of determining whether isolates are susceptible, intermediate or resistant, and to therefore guide appropriate antibiotic therapy in patients with an infectious disease. Importantly, this method has already been demonstrated to be robust and reproducible, and both quality control data and troubleshooting guides are readily available [31, 38]. The broth microdilution method has also been used to generate antimicrobial data for honey in numerous previous studies [21, 42, 51, 52]. Results from a selection of recent studies using methodology comparable to the current study, with similar bacterial test strains and examining Australasian honeys including Manuka honey are described here. For example, a previous study found MICs for six different monofloral honeys ranging from 3.12–25% against four *S*. *aureus* strains, and 6.25–25% for both *E*. *coli* and *P*. *aeruginosa* [21]. Similarly, MIC ranges for 11 monofloral honeys were 4–32% for *S*. *aureus*, 16–32% for both *E*. *coli* and *P*. *aeruginosa* and 16 - >32% for *E*. *faecalis* [32]. Girma et al (2019) tested multiple bacterial strains against three Manuka honeys and found the concentrations of honey at which 90% of strains were inhibited ranged from 7 to 15% for *S*. *aureus*, 21–27% for *P*. *aeruginosa* and was 33% for the Enterobacteriaceae [16]. MIC ranges from previous studies were similar to the current study, as was the overall trend whereby *S*. *aureus* was generally the most susceptible organism, followed by *E*. *coli* and *P*. *aeruginosa* which tended to have similar susceptibilities. Advantages of the broth microdilution assay are that it is robust and reproducible, generates quantitative data and is relatively easy to perform. Disadvantages are that the doubling dilutions typically employed in a broth microdilution method are usually insufficient for testing honey as the intervals between each sequential concentration may be relatively large. Alternative dilution series with smaller increments such as 2% [42] or 1% [53] are required, which increases both the labour intensity and the time required to perform the assay. Also, off-scale results may be encountered, depending on the range of honey concentrations tested. Lastly, the MIC endpoint, which is typically defined as the complete inhibition of growth, may not be easy to determine and does not necessarily capture all of the activity shown by honey. For example, depending on the concentration of honey tested, some honeys will show bacteriostatic activity whereby growth is reduced, but not completely prevented [2, 54]. The extent of this growth inhibition can be quantified by spectrophotometric optical density measurements [29], and this was utilised when developing the modified version of the broth microdilution assay to become the new antibacterial activity assay.

Spectrophotometric methods to measure optical density have been used previously for quantifying antibacterial activity [55, 56], including that of honey [57–59]. A highly relevant example of this is a paper by Patton et al (2006) that describes the development of a 96-well microtitre plate spectrophotometric assay for determining bacterial sensitivity to honey, adapted from previously published literature [29]. They found that their assay was simple and rapid, more sensitive than the standard disc or well diffusion assays and had the advantage of eliminating a subjective observation as the assay endpoint. They also found their assay to have good reproducibility and repeatability. Similar to the current study, the authors calculated percentage growth inhibition relative to the positive growth control for each organism, honey and concentration. They then used these data to plot dose-response curves and to determine the concentrations at which growth was inhibited by 100%, 50% and 0%, and several authors have since followed their methodology [30, 60]. This method provides valuable information about the susceptibility of individual organisms to honey, and shows that differences in optical density are a valid means of quantifying the antibacterial activity of honey. However, it is probably not easily adaptable to commercial use due to the level of data manipulation required and type of endpoint generated. The new assay described in the current work is essentially a modified broth microdilution assay, with an endpoint based on a similar principle to the test described by Patton et al., (2006) whereby percentage growth inhibition after incubation with honey is quantified. Advantages of the new assay compared to both the PE and MIC methods are that it is able to quantify activity over the entire range of honey activity (including those with relatively low activity), and has a non-visual, non-subjective optical density endpoint that does not rely on human interpretation. It also uses four test organisms, thus provides a broader representation of honey activity compared to data obtained using a single test species. Also, the PE assay is conducted over a four day period, whereas the new assay requires only three days. The new assay is not as high throughput as the PE assay (where up to 23 honeys can be tested in duplicate simultaneously), but offers more flexibility as individual honeys can be tested on demand.

Comparison of results from the new assay with both the PE results and the MIC results showed a reasonable level of agreement. Antibacterial activity values correlated strongly with MICs, which is perhaps to be expected given that these two assays have very similar methodologies. A lower degree of correlation was evident between results of the new assay and the PE assay, which can similarly be explained by the fundamental differences in methodology. A previous study examining the activity of 56 honey and honeydew samples using agar dilution, broth microdilution and agar diffusion showed that the agar and broth dilution methods gave similar results, whereas the relationship between agar diffusion and broth dilution results was not as straightforward [61]. Importantly, the authors noted that those samples with high activity in the broth assay showed a range of activities by agar diffusion, which they postulated was due to the presence of high molecular weight compounds with limited mobility in agar [61]. Similarly, a study with six honeys and four test bacteria showed that honeys showing highest activity by broth microdilution were not the same as those showing the highest activity by agar diffusion [35]. These previous findings support the conclusion that agar assays are not appropriate for quantifying the antibacterial activity of honeys.

A critical component of the development of any new laboratory assay is assay validation and verification. Guidelines are available to assist in validation studies [39], however, some parameters described within the guidelines apply to analyte detection assays, but are not applicable to antibacterial activity assays. The validation approach for antibacterial activity assays is therefore slightly different [62, 63]. Importantly, operator-dependent variables and operator-independent factors must be assessed and wherever possible, adequately controlled or minimised [64]. Assessment of the methodology described in the current paper showed that it had appropriate repeatability, reproducibility and robustness. In particular, validation by a second operator in a separate laboratory demonstrated that the assay had good reproducibility, which has been noted previously as a critical element of assay validation [65]. Once an assay has been adopted, ongoing quality control and participation in quality assurance programs are also vital [63].

Regardless of which antibacterial testing method is used, minor differences in the antibacterial activity of different honeys can be quantified in the laboratory. It remains to be determined whether these differences are reflected in clinical outcomes when treating disease. In the absence of this correlation between laboratory findings and clinical outcomes, it is important to not overstate, or extrapolate from results obtained in the laboratory. The new assay has the capacity to measure activity to single digits or activity units, however, small differences in activity of only a few units are unlikely to be statistically significantly different. Therefore, whilst reporting activity at the single-digit level is important for research purposes, it may lead to the over-interpretation of minor differences in results by both consumers and honey retailers. As such, it is recommended that a scale be created whereby antibacterial activity values are rounded to the nearest 50 or 100 units, and that the resulting antibacterial activity scale have a minimum of 100 to a theoretical maximum of 750. These are important parameters to establish, as in contrast, there do not appear to be any standard guidelines for how testing laboratories should report PE values, with some laboratories rounding values to the nearest five and others reporting to single digits. Also, the minimum and maximum values reportable using the PE assay do not appear to have been clearly established.

This study has developed and validated a new method for quantifying the antibacterial activity of honey, however, the study is not without limitations. For example, only honeys from a limited geographical area, and from limited floral sources were tested. If these honey samples were all relatively similar in antibacterial activity, physicochemical composition or provenance, then results of antibacterial activity assays may be “skewed” and may not represent the full spectrum of antibacterial activity found in honeys. Therefore, the examination of additional honey samples using this methodology is recommended, particularly those with mid-range activity as few of these were identified in the current study. Also, the assessment of additional honey samples from commercially important floral sources such as *Leptospermum* (Manuka), and of commercially available therapeutic products is recommended. In addition, the new assay is not as high throughput as the existing PE assay, meaning that the time required to set up the assay may be perceived as a limitation. ideally a multi-centre ring test should be performed where individual samples from a single honey are sent to different testing facilities to ascertain the degree of inter-laboratory agreement between testing results, and to obtain feedback on the usability of the new test.

In summary, a new assay has been developed that accurately quantifies the antibacterial activity of honeys, including those with relatively low activity. Adoption of the new testing protocol by industry participants, commercial testing laboratories and researchers would have wide-ranging benefits by providing a unified measurement of the antibacterial activity of all honeys.

## Supporting information

S1 Appendix (DOCX)
