# Supplementary material for: Development and validation of a new microplate assay that utilises optical density to quantify the antibacterial activity of honeys including Jarrah, Marri and Manuka
Source: PLoS One. 2020 Dec 9;15(12):e0243246. doi: 10.1371/journal.pone.0243246 (PMC7725308; doi:10.1371/journal.pone.0243246)
Supplement: S1 Appendix — (DOCX) [file pone.0243246.s001.docx]

**Supplementary Table 1.** Instrument quantitation studies comparing theoretical and actual cell density measurements.

|  | *S. aureus* | | *E. coli* | | *E. faecalis* | | *P. aeruginosa* | |
| --- | --- | --- | --- | --- | --- | --- | --- | --- |
| Theoretical | Actual | Difference | Actual | Difference | Actual | Difference | Actual | Difference |
| 100 | 100.0 | 0.0 | 100.0 | 0.0 | 100.0 | 0.0 | 100.0 | 0.0 |
| 90 | 97.3 | 7.3 | 95.6 | 5.6 | 89.0 | -1.0 | 96.3 | 6.3 |
| 80 | 86.6 | 6.6 | 85.1 | 5.1 | 80.2 | 0.2 | 90.1 | 10.1 |
| 70 | 77.9 | 7.9 | 75.5 | 5.5 | 70.4 | 0.4 | 82.0 | 12.0 |
| 60 | 69.9 | 9.9 | 66.2 | 6.2 | 61.6 | 1.6 | 72.4 | 12.4 |
| 50 | 61.4 | 11.4 | 56.3 | 6.3 | 51.2 | 1.2 | 62.2 | 12.2 |
| 40 | 50.3 | 10.3 | 46.3 | 6.3 | 41.9 | 1.9 | 52.3 | 12.3 |
| 30 | 38.2 | 8.2 | 37.4 | 7.4 | 32.5 | 2.5 | 40.9 | 10.9 |
| 20 | 29.7 | 9.7 | 25.0 | 5.0 | 23.4 | 3.4 | 26.6 | 6.6 |
| 10 | 15.2 | 5.2 | 13.3 | 3.3 | 12.9 | 2.9 | 13.6 | 3.6 |
| Mean |  | 7.65 |  | 5.08 |  | 1.32 |  | 8.63 |

**Supplementary Table 2.** Optimisation of assay incubation period, determined by measuring optical density values for various conditions at several time points. Note decreases or increases in optical density between 22 and 24 h.

| Conditions | Organism | 18 h | 20 h | 22 h | 24 h |
| --- | --- | --- | --- | --- | --- |
| Control wells | *S. aureus* | 0.630 | 0.700 | 0.751 | 0.885 |
| (no honey) | *E. coli* | 0.577 | 0.621 | 0.690 | 0.309 |
|  | *E. faecalis* | 0.366 | 0.379 | 0.380 | 1.053 |
|  | *P. aeruginosa* | 0.931 | 0.980 | 0.998 | 0.371 |
|  |  |  |  |  |  |
| Pasture honey | *S. aureus* | 0.419 | 0.418 | 0.432 | 0.509 |
| (10% w/v) | *E. coli* | 0.523 | 0.505 | 0.506 | 0.394 |
|  | *E. faecalis* | 0.374 | 0.365 | 0.360 | 1.228 |
|  | *P. aeruginosa* | 1.245 | 1.276 | 1.235 | 0.085 |


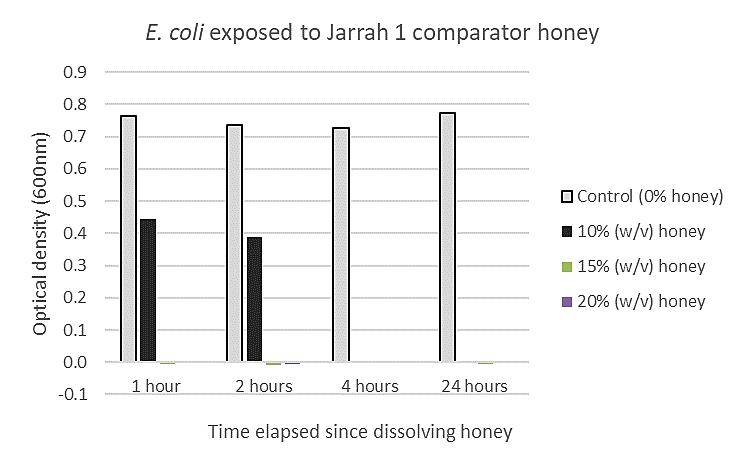


Supplementary Figure 1: *E. coli* exposed to Jarrah 1 comparator honey. Honey showed higher activity after 4 h, compared to both 1 h and 2 h.
